# Awake Tracheal Intubation Is Associated with Fewer Adverse Events in Critical Care Patients than Anaesthetised Tracheal Intubation

**DOI:** 10.3390/jcm12186060

**Published:** 2023-09-19

**Authors:** Marc Kriege, Rene Rissel, Hazem El Beyrouti, Eric Hotz

**Affiliations:** 1 Department of Anaesthesiology, University Medical Centre of the Johannes Gutenberg-University, 55131 Mainz, Germany; 2 Department of Cardiac and Vascular Surgery, University Medical Centre of the Johannes Gutenberg-University, 55131 Mainz, Germany

**Keywords:** videolaryngoscopy, intensive care, tracheal intubation, complication

## Abstract

Background: Tracheal intubation in critical care is a high-risk procedure requiring significant expertise and airway strategy modification. We hypothesise that awake tracheal intubation is associated with a lower incidence of severe adverse events compared to standard tracheal intubation in critical care patients. Methods: Records were acquired for all tracheal intubations performed from 2020 to 2022 for critical care patients at a tertiary hospital. Each awake tracheal intubation case, using a videolaryngoscope with a hyperangulated blade (McGrath^®^ MAC X-Blade), was propensity matched with two controls (1:2 ratio; standard intubation videolaryngoscopy (VL) and direct laryngoscopy (DL) undergoing general anaesthesia). The primary endpoint was the incidence of adverse events, defined as a mean arterial pressure of <55 mmHg (hypotension), SpO_2_ < 80% (desaturation) after sufficient preoxygenation, or peri-interventional cardiac arrest. Results: Of the 135 tracheal intubations included for analysis, 45 involved the use of an awake tracheal intubation. At least one adverse event occurred after tracheal intubation in 36/135 (27%) of patients, including awake 1/45 (2.2%; 1/1 hypotension), VL 10/45 (22%; 6/10 hypotension and 4/10 desaturation), and DL 25/45 (47%; 10/25 hypotension, 12/25 desaturation, and 3/25 cardiac arrest; *p* < 0.0001). Conclusions: In this retrospective observational study of intubation practices in critical care patients, awake tracheal intubation was associated with a lower incidence of severe adverse events than anaesthetised tracheal intubation.

## 1. Introduction

Tracheal intubation in critical care patients may be associated with severe adverse peri-intubation events [1,2,3]. The “physiologically difficult airway” describes physiologic derangements, including hypoxemia, hypotension, severe metabolic acidosis, and right ventricular failure [4]. Prospective studies and retrospective analyses have shown that life-threatening complications can occur among critical care patients undergoing tracheal intubation, such as hemodynamic instability in 42.6% of patients [1], severe hypoxemia (SpO_2_ < 80%) in 9–25% of patients [1,2], and cardiac arrest in 2.7–3.1% of patients [1,2,5]. The failure of first-pass intubation occurs in up to 30% of tracheal intubations in the ICU [6,7,8] compared to the OR.

Awake tracheal intubation is the common standard for the management of the anticipated difficult airway in adult patients [9,10,11,12]. Traditionally, this has been accomplished using a fibreoptic endoscope or a videolaryngoscope (VL) [13,14,15,16]. Awake videolaryngoscopy probably seems to be faster than, and at least as safe as, awake fibreoptic intubation. However, there are several unanswered questions about the role of awake videolaryngoscopy in the field of airway management: could it be used instead of rapid sequence induction in patients with a high risk of aspiration or in patients with an anatomical or physiologically difficult airway instead of using general anaesthesia? There are several studies comparing awake videolaryngoscopy with awake fibreoptic intubation [14,15,17,18]. Studies evaluating patients with awake videolaryngoscopy compared to anaesthetised patients using videolaryngoscopy or direct laryngoscopy do currently not exist.

To the best of our knowledge, no studies or reports have described the incidence of adverse events associated with awake tracheal intubation using videolaryngoscopy in critical care patients. The aim of this study was to determine the difference in incidences of severe peri-intubation events between awake tracheal intubation and standard intubation. We hypothesise that awake tracheal intubation is associated with a lower incidence of severe adverse events than standard tracheal intubation in critical care patients.

## 2. Materials and Methods

### 2.1. Ethical Consideration

The local ethics committee (Ethical Committee No. 2019-14651) of the medical association of Rhineland-Palatinate state (Chair Dr. A. Wagner), Germany, approved this study (approval date: 31 October 2019; Study Title: Evaluation of Airway management in critical care patients in a tertiary hospital; NCT 05802316). Data were fully anonymised before researchers accessed them, so written informed patient content was not necessary and was waived by the ethics committee. This study followed the guidelines of the Declaration of Helsinki.

### 2.2. Study Design

**Figure 1:**
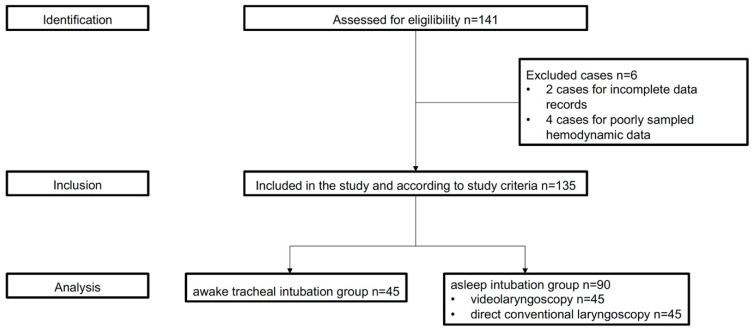
CONSORT diagram illustrating the flow of patients in the study.

This study was performed in accordance with the Strengthening the Reporting of Observational Studies in Epidemiology (STROBE) statement [19]. This was a retrospective matched cohort single-centre study of data from critical care patients requiring tracheal intubation from 2020 to 2022. The records of all patients were retrieved and reviewed by the author’s group (Figure 1).

### 2.3. Inclusion and Exclusion Criteria

Data for all critical care patients requiring tracheal intubation using awake videolaryngoscopy, or videolaryngoscopy and direct laryngoscopy undergoing rapid sequence induction at the medical ward, ER, or ICU during the study period were included. Exclusion criteria included incomplete data reports and patients requiring tracheal intubation during cardiopulmonary resuscitation.

### 2.4. Data Collection Procedure

A data extraction sheet was used to obtain information on the demographic profile of each patient, the tracheal intubation technique, vital signs, and complications using the local patient data management system (Copra^®^ System RM 1.0 GmbH, Berlin, Germany). These data are passively and routinely collected by the Copra System, which records vital signs from the monitor in one-minute intervals. Each intubation procedure was recorded immediately after tracheal intubation (e.g., indication, medication, preoxygenation, performance, success, complications) parallel to the vital sign from the patient data management system. Propensity matching included (i) patient characteristics (e.g., age, sex, ASA status, BMI); (ii) medical conditions (e.g., arterial hypertension, coronary artery disease); (iii) MACOCHA score [3]; and (iv) the level of training. To determine the potential bias introduced by the large number of cases excluded for inaccurate or incomplete records, the primary and secondary outcomes were also measured for cases excluded for those reasons for comparison. The study group reviewed each chart individually to minimise inaccurate descriptions and omissions and to determine whether any factors existed that would lead to the exclusion of a case from analysis, or whether there were complications associated with the intubation procedure. The comprehensive chart for each awake videolaryngoscopy underwent an initial review by at least one of the authors and was flagged for further review if any complication appeared to be present based on available intraoperative documentation. Additionally, an independent nurse who was not involved in the study confirmed that the individual data extractions were complete.

### 2.5. Study Setting

This study was carried out from January 2020 to April 2022 at the University Medical Centre Mainz, Germany, which is a tertiary hospital. We chose this time range based on the start date of the awake videolaryngoscopy teaching program in our tertiary hospital. Based on the records obtained, two groups were established for the same time period: the awake tracheal intubation using videolaryngoscopy group (awake); and the anaesthetised intubation group (anaesthetised) using a VL or direct conventional laryngoscope (DL). The choice of awake tracheal intubation or anaesthetised intubation was at the discretion of the provider and based on patient assessment (anatomically or physiologically difficult airway).

All awake tracheal intubations were performed or supervised by an experienced anaesthesiologist. Tracheal intubation in the anaesthetised group was performed by an expert, defined as a physician who had either worked in the ICU for at least 5 years or a physician who had worked in the ICU for at least 1 year after receiving at least 5 years of anaesthesiology training. Physicians who did not meet these criteria were classified as nonexperts (ICU experience of between 3 and 12 months). All physicians had previous training in airway management with a simulator (>75 times previously for direct laryngoscopy and >25 times previously for videolaryngoscopy) and in real patients in the operating room (>25 times previously for direct laryngoscopy and >10 times previously for videolaryngoscopy). The McGrath^®^ MAC (Medtronic^®^, Brampton, ON, Canada) with a size 3 or 4 Macintosh-based blade was used for videolaryngoscopic intubation. For the awake group, a hyperangulated X-blade was used. A standard laryngoscope (Heine^®^ Optotechnik, Gilching, Germany) using a size 3 or 4 Macintosh blade was used for DL. For both groups, the trachea was intubated with a tracheal tube size of 7.5 or 8.0 mm and a malleable stylet (Mallinckrodt^TM^ style) at a 90° angle, 8 cm from the distal tip [20].

All patients were monitored using ECG, non-invasive or invasive blood pressure monitoring, and pulse oximetry. In the awake group, patients were administered oxygen through a high-flow nasal cannula with a flow rate of 60 L per minute and a fraction of inspired oxygen of 1.0. Topical anaesthesia was applied using lidocaine 10% metered spray (10 mg per puff) nebulised through a mouthpiece directly on the mucosa of the oropharynx. To avoid toxic reactions, a maximum lidocaine dose of 9 mg·kg^−1^ lean body weight was adopted [21]. The sufficiency of the pharyngeal and laryngeal analgesia was evaluated by the patients’ acceptance before the subsequent tracheal topicalisation. In addition, a transtracheal injection (MADgic^®^ Laryngo-Tracheal Mucosal Atomization Device, Teleflex^®^, Wayne, PA, USA) of 30 mg lidocaine in a maximum volume of 3 mL was administered. Before airway manipulation, each patient received an initial dose of 1.0 µg·kg^−1^ dexmedetomidine over 10 min followed by 0.8 µg·kg^−1^, both by continuous infusion. To avoid over-sedation with a potential airway obstruction, a Ramsay sedation score of 2 (patient co-operative) was not exceeded [22]. An intubation attempt was considered unsuccessful if the tracheal tube was removed to the oral cavity due coughing, gagging, or the inability to view the vocal cords.

All patients in the anaesthetised group were preoxygenated with 100% oxygen (e.g., standard face mask, bag valve mask, non-invasive ventilation, or high-flow nasal cannula). Anaesthesia was induced following rapid sequence induction and neuromuscular blocking agent as part of their induction. The laryngoscopy attempt in the anaesthetised group was defined as successful if the tracheal tube was placed (until the black mark on the tracheal tube was threaded between the vocal cords) with a single blade insertion within 120 s and without the manipulation of the laryngoscope by another provider.

### 2.6. Outcome

The primary outcome measured herein was the incidence of adverse peri-intubation events, defined as the occurrence of at least one of the following events within 30 min from the start of the tracheal intubation procedure: (1) mean arterial pressure of <55 mmHg (hypotension) for >30 min; (2) severe hypoxemia (oxygen saturation as measured by pulse oximetry SpO_2_ < 80%; desaturation) after sufficient preoxygenation (SpO_2_ > 98%); and (3) peri-interventional cardiac arrest. The secondary outcomes included the first-pass intubation success (FPS) rate, laryngeal visualisation with the Cormack & Lehane (C&L) classification, and peri-interventional complications (e.g., aspiration of gastric contents, oesophageal intubation, airway injury such as dental injury or soft tissue lesions). We also documented the skill level technique, anaesthesia medications, and confirmation of tracheal intubation.

### 2.7. Statistical Analysis

All collected data were analysed using GraphPad Prism 9.0 h (GraphPad^®^ Software Inc., San Diego, CA, USA). The Shapiro–Wilk test was used to examine the distribution of each variable. Normally distributed variables are presented as the mean and standard deviation (SD), while nonnormally distributed variables are presented as the median and interquartile range (IQR). For comparisons between the awake and anaesthetised groups, the chi-square or Fisher exact test was used for categorical variables, and Student’s *t*-test or the Mann-Whitney test was used for continuous variables, as appropriate. Statistical significance was set at a *p* value of <0.05. Propensity matching was used to construct similar sets of anaesthetised patients and awake intubation patients. This process enabled us to account for various factors that could affect peri-intubation events. Specifically, we developed a logistic regression model to predict the propensity for receiving awake intubation using pre-intubation variables, including age, sex, body mass index (BMI), American Society of Anesthesiologists (ASA) physical status classification, level of training, MACOCHA score, and whether the patient had co-morbidities (e.g., obstructive sleep apnoea syndrome (OSAS), chronic kidney disease, end-stage renal disease, arterial hypertension, congestive heart failure (CHF), chronic obstructive pulmonary disease, coronary artery disease (CAD), and atrial fibrillation or flutter (Afib)). Furthermore, we include factors which have a direct impact on the outcome measures such as pulmonary disease (e.g., aspiration pneumonitis, pneumothorax, lung atelectasis) and shock state (e.g., hypovolemic shock, cardiogenic shock).

Next, we paired awake and anaesthetised subjects in a 1:2 ratio based on propensity scores using nearest neighbour matching without replacement. Calliper matching was not used. The balance between the two groups was carefully examined based on the standardised mean differences between groups (Table 1). The analysis was performed using the MatchIt package in R 3.2.3 (The R Foundation for Statistical Computing^®^, Vienna, Austria).

To determine this potential bias introduced by the large number of cases excluded for inaccurate or incomplete records, the primary and secondary outcomes were also measured for cases excluded for those reasons for comparison. The study group reviewed each chart individually to minimise inaccurate descriptions and omissions and to determine whether any factors existed that would lead to the exclusion of a case from analysis, or whether there were complications associated with the intubation procedure. The comprehensive chart for each awake videolaryngoscopy underwent an initial review by at least one of the authors and was flagged for further review if any complication appeared to be present based on available intraoperative documentation. Additionally, an independent nurse who was not involved in the study confirmed that the individual data extractions were complete.

## 3. Results

Between 2020 and 2022, 149 patients were assessed for eligibility. Before propensity matching, 4 of the 49 patients in the awake intubation group (8.2%) and 2 of the 92 patients in the anaesthetised intubation group (2.2%) were excluded. Eight patients with tracheal intubation during cardiopulmonary resuscitation were also excluded. The majority of exclusions were due to incomplete records. A total of 135 patients were enrolled in the study (Table 1). Within the propensity score-matched population, there were no significant peri-intubation factors associated with the incidence of adverse events.

Of the critical care patients undergoing tracheal intubation, at least one adverse event occurred in 36/135 (27%) patients (Table 2). There was a significantly higher incidence of adverse events 35/90 (39%) in the anaesthetised group compared with the awake group 1/45 (2.2%; *p* < 0.0001). Hypotension was the most frequent adverse event in the anesthetised group (16/90, 18%), compared to the awake group (1/45, 2.2%; *p* < 0.0001). From baseline, one patient in the awake group and four patients in the anaesthetised group had cardiogenic shock, eight patients in the anaesthetised group had respiratory failure, and four patients had an impairment of the neurological status. In the anesthetised group, desaturation was more frequent (12/45, 27%) using DL (*p* < 0.002), compared with VL (4/45, 8.8%).

The primary outcome was more frequently observed when nonexperts (31/36, 86.1%) performed tracheal intubation than when experts (5/36, 13.9%) performed tracheal intubation. This resulted in a relative risk of adverse events of 0.16 (95% [CI 0.07–0.33], *p* < 0.0001) when experts performed tracheal intubation compared to nonexperts. However, in the awake group when nonexperts performed tracheal intubation, the incidence of adverse events was less frequent compared to the anaesthetised group (*p* < 0.0001). A MACOCHA score of ≥4 was associated with a higher incidence of adverse events (35/36, 97.2%) and a relative risk of 0.02 (95% [CI 0.005–0.146], *p* < 0.0001), as compared to a MACOCHA score of ≤3.

Regarding the secondary outcomes, there was a significantly higher FPS rate in the awake group (45/45, 100%) than in the anaesthetised group (71/90, 78.9%; *p* < 0.001). Table 3 shows glottis visualisation, alternative techniques for failed intubation, medications, and confirmation of intubation. In the awake group, for adequate topical anaesthesia we used a median dose of 5.4 mg·kg^−1^ (3.8–6.2 mg·kg^−1^) lidocaine. A laryngeal view with clear view of the vocal cords (C&L grade I or II) was observed more frequently in the awake group. When nonexperts performed laryngoscopy, glottic visualisation with a C&L grade ≥III was more frequent in the anaesthetised group (8/90, 8.8%) than in the awake group (0/45, 0%) (*p* = 0.003). The location of intubation is shown in Table 3.

Resident physicians performed the vast majority of intubations (123/135; 91%; Table 3), and every case was supervised by a consultant who had performed more than 25 awake tracheal intubations with the VL or more than 1000 tracheal intubations with DL. Anaesthesiologist residents performed intubation in 108/135 (80%) of the cases, while the other physicians were emergency physicians (10/135, 7.4%) or cardiac surgery physicians (5/135, 3.7%).

## 4. Discussion

In this retrospective matched cohort single-centre study, we observed a lower incidence of adverse events in critical care patients when using the awake tracheal intubation technique with VL (2.2%) than when using anaesthetised tracheal intubation (39%). To the best of our knowledge, this is the first study investigating awake tracheal intubation with a videolaryngoscope compared to standard intubation in critical care patients.

Of all the reported tracheal intubations, the incidence of adverse events was 27%. The results of the primary outcome were comparable to those of two previous studies, which reported that at least one severe complication occurred in 28% [23] and 29.8% [24] of tracheal intubations. In our study, the reasons for the increased incidence of adverse events included a second or third attempt, insufficient glottic visualisation using the DL, or difficult tracheal tube insertion through the vocal cord level despite sufficient glottic visualisation in the anaesthetised group with videolaryngoscopy.

The multicentre INTUBE study showed that among 2964 critical care patients, 45.2% experienced at least one adverse peri-intubation event [1]. Furthermore, in this multivariable analysis, several patient- and setting-related variables were identified. One other study reported an association between the use of the VL with a Macintosh blade and a higher rate of severe life-threatening complications compared to DL [25]; however, this study included more nonexperts and fewer anaesthesiologists than our current study, and the median intubation time for successful intubation was 3 min for both VL and DL.

The role of videolaryngoscopy in facilitating tracheal intubation in critical care patients remains unclear. A recently published meta-analysis of randomised studies comparing VL with DL for intubation in critical care patients showed that VL did not shorten the time to intubation or improve FPS, irrespective of the operator’s experience [26]. Other studies have shown that videolaryngoscopy improved difficult intubation, FPS, C&L grade 3 or 4, and accidental oesophageal tracheal intubation and did not modify severe hypoxemia, severe hemodynamic instability, or airway injury compared with DL [24,27,28]. However, there are multiple possible reasons for these heterogeneous results: (i) The main challenge with the videolaryngoscopy is the insertion of the tracheal tube into the trachea rather than the visualisation of the glottis. Achieving a 100% glottis opening view (corresponding to a C&L grade 1) during videolaryngoscopy does not guarantee successful placement of the tracheal tube [29]. (ii) Inexperienced operators or non-anaesthesiologists tend to lose time when attempting tracheal tube insertion under indirect visualisation, thereby increasing the time to intubation, which is associated with increased peri-intubation-related complications, such as severe hypoxemia. Furthermore, there is no universal ideal VL, and each laryngoscope or blade design (Macintosh vs. hyperangulated) has its own characteristics, strengths, weaknesses, and learning curve [30,31]. Awake tracheal intubation using videolaryngoscopy has a comparable success rate and safety profile compared to flexible fibreoptic tracheal intubation (98% each) [18,32,33,34]. Experience with awake tracheal intubation using VL in the ICU or data comparing awake videolaryngoscopy with anaesthetised patients in the OR is lacing. However, studies comparing awake videolaryngoscopy with awake fibreoptic intubation have shown differences in the term “awake”. All of these studies used remifentanil infusion with or without midazolam boluses as sedation for both awake videolaryngoscopy and awake fibreoptic intubation [14,15,17,18,35,36]. All intubations were performed under topical anaesthesia and moderate sedation (e.g., target Ramsay score of 3 to 5) with spontaneous breathing. This raises the question how “awake” patients undergoing these intubation procedures truly are. A Ramsay sedation score describes levels 1 to 3 as “awake” and 4 to 6 as “anaesthetised”. The pharmacological side effect during induction of general anaesthesia is that patients may exhibit significant decreases in heart rate or blood pressure, especially in critical ill patients with a reduced left ventricular ejection fraction or hypovolemia. So, deeper sedation may reduce the safety margin offered by nominally “awake” intubation techniques. In terms of patients comfort and experience, adequate topical anaesthesia is vital for the success of any “awake” technique; there is still uncertainty about the needed level of sedation or Ramsay sedation score required to make awake videolaryngoscopy-guided intubation acceptable for patients [37]. However, we hypothesised that the acceptance and success of the awake intubation method is likely to be influenced more by the adequacy of topical anaesthesia than the level of sedation.

The FPS rates in the anaesthetised group (89% with the VL and 69% with the DL) were comparable with the previously reported data in critical care patients with VL and DL [16,38,39]. Several studies have shown that the risk of complications increases with prolonged intubation attempts. Consequently, a higher FPS when using a VL should be associated with fewer complications [6,40,41].

In regard to an improved C&L grade of view and reduced risk of difficult intubation and oesophageal intubation, several studies revealed similar results to our study [1,25,41,42,43].

Furthermore, we observed more adverse events when nonexperts performed tracheal intubation than when experts performed tracheal intubation. The distribution of level of experience was comparable to other studies including experts and nonexperts [1,16,25,38,39]. We should consider that expertise in DL use does not automatically translate into a high success rate using a VL. In one study, expert users of DL needed 76 attempts to achieve similar expertise with a VL [44]. This might also explain the results of a multi-centre study in critical care patients, which did not show a difference between VL and DL [45]. In this trial, other adverse events and airway injuries were observed in 0% of patients in the awake group and 16.6% of patients in the anaesthetised group, comparable to other studies examining expected low-risk airway management [16,25,38,39,46]. Notably, the increased incidence of airway injuries was associated with ≥2 tracheal intubation attempts. This is reinforced by data demonstrating increased oropharyngeal or dental injuries with multiple intubation attempts [46,47,48,49].

There are several approaches to reduce peri-intubation complications, but the evidence to achieve cardiovascular stability before tracheal intubation is currently limited. Two trials investigated the effectiveness of a bundle of interventions to reduce peri-intubation adverse events, and the authors reported that its implementation was associated with a reduction in cardiovascular collapse and severe hypoxemia compared with their incidence registered during the baseline period [50,51].

## 5. Limitations

This trial has several limitations. First, the study design relied on the physician documenting the type of intubation (awake vs. anaesthetised), the device used to intubate (VL vs. DL), and details regarding complications. It is possible that some awake intubations performed during the study period were not identified due to poor documentation, and the same is true regarding complications and failures. Second, the findings may not be generalisable to the prehospital setting or OR. This study assessed the use of a single VL with a Macintosh blade or hyperangulated blade; thus, the results might not be transferable to other available videolaryngoscopes, particularly those using channelled blades. Additionally, we did not compare awake videolaryngoscopy with awake fibreoptic intubation. Third, in regard to the retrospective study design, we cannot evaluate the performance of anaesthetists with differing degrees of experience with tracheal intubation. Fourth, we identified no interventions aiming to achieve cardiovascular stability before tracheal intubation. Fifth, we cannot guarantee that awake intubation was “somehow like at random”.

Finally, this study did not collect information on the direct long-term consequences of acute versus peri-intubation events on specific patient outcomes (e.g., hypoxic brain injury) and selection bias in this retrospective population makes it very difficult to interpret the results (patients selected for anaesthetised tracheal intubation or not).

## 6. Conclusions

In conclusion, this retrospective observational study provides evidence that awake tracheal intubation using a videolaryngoscope in critical care patients helps to reduce adverse peri-intubation events, increase the FPS rate, optimise glottis visualisation, and reduce airway injury compared to VL and DL in anaesthetised critical care patients.

## Figures and Tables

**Table 1:** Patient characteristics. Values are presented as the means, standard deviations (SDs), and relative proportions (%).

	All Patients *n* = 135	Awake Group *n* = 45	Anaesthetised Group *n* = 90	SMD
**Age (y)**	59.61 (8.9)	60.4 (9.60)	59.21 (8.63)	−0.1304
**Sex**				
Male	69 (51%)	23 (51.1%)	46 (51.1%)	0.0005
Female	66 (49%)	22 (48.9%)	44 (48.9%)	
**ASA status**				
III	29 (21%)	10 (22.2%)	19 (21.2%)	0.0092
IV	96 (71%)	32 (71.1%)	64 (71%)	
V	10 (8%)	3 (6.7%)	7 (7.8%)	
**BMI (kg·m^2^)**	26.13 (4.43)	25.36 (3.8)	26.51 (4.69)	−0.071
**Comorbidities**				
Has OSAS	11 (8.1%)	4 (8.8%)	7 (7.7%)	0.0115
Has CKD	17 (12.6%)	6 (13.3%)	11 (12.2%)	0.012
Has arterial Hypertension	85 (62.9%)	22 (44.5%)	63 (46.6%)	−0.005
Has CHF	46 (34%)	15 (33.3%)	31 (34.4%)	0.0034
Has COPD	23 (25.5%)	8 (17.7)	15 (16.6%)	−0.002
Has CAD	16 (11.8%)	5 (11.1%)	11 (12.2%)	0.0115
Has AFiB	18 (20%)	6 (13.3%)	12 (13.3%)	0.0005
**ICU related factors**				0.003
Pulmonary disease	28 (21%)	10 (22%)	18 (20%)	
Shock state	25 (18%)	9 (20%)	16 (18%)	
**MACOCHA score**				
3	49 (36%)	15 (33.3%)	34 (37.8%)	0.0023
4	60 (44.3%)	19 (42.2%)	41 (45%)	
5	17 (12.6%)	4 (8.9%)	13 (14%)	
6	8 (6%)	6 (13.3%)	2 (2.2%)	
7	1 (1.1%)	1 (2.3%)	0	
**Level of training**				
nonexperts	123 (91%)	45 (100%)	78 (87%)	0.06
experts	12 (9%)		12 (13%)	

**Abbreviations:** ASA (American Society of Anesthesiologists), BMI (body mass index), OSAS (obstructive sleep apnoea syndrome), CKD (chronic kidney disease), CHF (chronic heart failure), CAD (coronary artery disease), AFiB (atrial fibrillation). MACOCHA score has been developed to predict intubation failure of non anaesthesiologist intensive care unit trainees (defined as a doctor with less than 2 years of anaesthesiological training/nonanaesthesiologist intensivists). The calculation includes Mallampati scores of III and IV (5 points), OSAS (2 points), reduced mobility of the cervical spine (1 point), limited mouth opening less than 3 cm (1 point), coma (1 point), severe hypoxemia (1 point), nonanaesthesiologist operator (1 point) (range from 0 (easy) to 12 (very difficult)) [3], and SMD (standard mean difference between awake and matched anaesthetised group).

**Table 2:** Outcome parameters. Values are presented as absolute numbers and relative proportions (%).

Adverse Event	All Patients *n* = 135	Awake Group *n* = 45	Anaesthetised Group *n* = 90		*p* Value
			VL *n* = 45	DL *n* = 45	
**Adverse event**	36 (27%)	1 (2.2%)	10 (22%)	25 (47%)	<0.0001
Hypotension	17 (12.6%)	1 (2.2%)	6 (13.3%)	10 (22%)	
Desaturation	16 (11.8%)	0 (0%)	4 (8.8%)	12 (27%)	
Cardiac arrest	3 (2.2%)	0 (0%)	0 (0%)	3 (6.6%)	
**Other adverse events**	8 (5.9%)	0 (0%)	3 (6.6%)	5 (11%)	0.08
Aspiration of gastric contents	4 (2.9%)	0 (0%)	1 (2.2%)	3 (6.6%)	
Oesophageal intubation	3 (2.2%)	0 (0%)	1 (2.2%)	2 (4.4%)	
**Airway injury**	7 (5.2%)	0 (0%)	1 (2.2%)	6 (13.3%)	0.009
Dental injury	2 (1.5%)	0 (0%)	0 (0%)	2 (4.4%)	
Soft tissue lesions	5 (3.7%)	0 (0%)	1 (2.2%)	4 (8.8%)	

**Table 3:** Peri-intubation variables. Values are presented as the means, standard deviations (SDs), and relative proportions (%).

Variables	Awake Group *n* = 45	Anaesthetised Group *n* = 90		*p* Value
		VL *n* = 45	DL *n* = 45	
**Main reason for tracheal intubation**				
Respiratory failure	26/45 (57%)	28/45 (62%)	20 (44%)	
Neurological impairment	14/45 (31%)	18/45 (40%)	14/45 (31%)	
Cardiovascular instability	4/45 (8.8%)	7/45 (15%)	3/45 (6%)	
**Preoxygenation method**				
Standard face mask	0 (0%)	10 (22%)	16 (36%)	0.16
Bag valve mask	0 (0%)	28 (62%)	19 (42%)	0.05
Non-invasive ventilation	0 (0%)	7 (16%)	10 (22%)	0.41
High-flow nasal cannula	45 (100%)	0 (0%)	0 (0%)	
**Anaesthetics**				
Topical anaesthesia	45 (100%)	0 (0%)	0 (0%)	
Dexmedetomidine	45 (100%)	0 (0%)	0 (0%)	
Propofol	0 (0%)	37 (82%)	33 (73%)	0.31
Ketamine	1 (2.2%)	8 (18%)	12 (27%)	1.04
Midazolam	0 (0%)	8 (18%)	12 (27%)	1.02
Sufentanil	0 (0%)	37 (82%)	33 (73%)	0.31
**Muscle relaxant**				
Succinylcholine	0 (0%)	5 (11%)	7 (16%)	0.51
Rocuronium	0 (0%)	40 (89%)	38 (84%)	0.53
**Method of laryngoscopy**				
Direct laryngoscopy	0 (0%)	13 (29%)	45 (100%)	<0.0001
Indirect videolaryngoscopy Macintosh blade	0 (0%)	25 (56%)	0 (0%)	
Videolaryngoscopy Hyperangulated blade	45 (100%)	7 (15%)	0 (0%)	<0.001
No. of attempts *				
First attempt	45 (100%)	40 (89%)	31 (69%)	<0.0001
Second attempt	0 (0%)	4 (8.8%)	11 (24%)	0.04
Overall success rate	0 (0%)	1 (2.2%)	3 (7%)	0.3
**Glottic view**				
C&L grade I/II/III/IV	43/2/0/0	39/4/2/0	23/16/4/2	0.001
**Alternative techniques**				0.78
SGA	0 (0%)	2 (4.4%)	1 (2.2%)	
Videolaryngoscope	0 (0%)	0 (0%)	1 (2.2%)	
eFONA	0 (0%)	0 (0%)	2 (4.4%)	
**Confirm intubation**				
Waveform capnography	45 (100%)	35 (78%)	39 (87%)	0.004
Auscultation	40 (89%)	45 (100%)	45 (100%)	0.005
**Level of experience**				
Nonexperts	45 (100%)	35 (78%)	43 (95%)	0.15
Experts	0	10 (22%)	2 (5%)	0.01
**Location of intubation**				
ICU	40 (89%)	39 (87%)	44 (97.8%)	
Emergency department	4 (8.8%)	6 (13%)	1 (2.2%)	
Medical ward	1 (2.2%)			

**Abbreviations:** C&L (Cormack & Lehane), SGA (supraglottic airway), eFONA (emergency front of neck access); ICU (Intensive Care Unit). * Reasons for a second or third attempt were a prolonged intubation time, insufficient glottic visualisation, or difficult tracheal tube insertion through the vocal cord level despite sufficient glottic visualisation in the anaesthetised group with videolaryngoscopy.

## Data Availability

The raw data supporting the conclusions of this article will be made available by the authors without undue reservation.

## References

[1] Russotto V., Myatra S.N., Laffey J.G., Tassistro E., Antolini L., Bauer P., Lascarrou J.B., Szuldrzynski K., Camporota L., Pelosi P. (2021). Intubation Practices and Adverse Peri-intubation Events in Critically Ill Patients from 29 Countries. JAMA.

[2] Jaber S., Amraoui J., Lefrant J.Y., Arich C., Cohendy R., Landreau L., Calvet Y., Capdevila X., Mahamat A., Eledjam J.J. (2006). Clinical practice and risk factors for immediate complications of endotracheal intubation in the intensive care unit: A prospective, multiple-center study. Crit. Care Med..

[3] De Jong A., Molinari N., Terzi N., Mongardon N., Arnal J.M., Guitton C., Allaouchiche B., Paugam-Burtz C., Constantin J.M., Lefrant J.Y. (2013). Early identification of patients at risk for difficult intubation in the intensive care unit: Development and validation of the MACOCHA score in a multicenter cohort study. Am. J. Respir. Crit. Care Med..

[4] Mosier J.M., Joshi R., Hypes C., Pacheco G., Valenzuela T., Sakles J.C. (2015). The Physiologically Difficult Airway. West. J. Emerg. Med..

[5] Griesdale D.E., Bosma T.L., Kurth T., Isac G., Chittock D.R. (2008). Complications of endotracheal intubation in the critically ill. Intensive Care Med..

[6] Mort T.C. (2004). Emergency tracheal intubation: Complications associated with repeated laryngoscopic attempts. Anesth. Analg..

[7] Schwartz D.E., Matthay M.A., Cohen N.H. (1995). Death and other complications of emergency airway management in critically ill adults. A prospective investigation of 297 tracheal intubations. Anesthesiology.

[8] Martin L.D., Mhyre J.M., Shanks A.M., Tremper K.K., Kheterpal S. (2011). 3,423 emergency tracheal intubations at a university hospital: Airway outcomes and complications. Anesthesiology.

[9] Apfelbaum J.L., Hagberg C.A., Connis R.T., Abdelmalak B.B., Agarkar M., Dutton R.P., Fiadjoe J.E., Greif R., Klock P.A., Mercier D. (2022). 2022 American Society of Anesthesiologists Practice Guidelines for Management of the Difficult Airway. Anesthesiology.

[10] Piepho T., Cavus E., Noppens R., Byhahn C., Dorges V., Zwissler B., Timmermann A. (2015). S1 guidelines on airway management: Guideline of the German Society of Anesthesiology and Intensive Care Medicine. Anaesthesist.

[11] Law J.A., Duggan L.V., Asselin M., Baker P., Crosby E., Downey A., Hung O.R., Jones P.M., Lemay F., Noppens R. (2021). Canadian Airway Focus Group updated consensus-based recommendations for management of the difficult airway: Part 1. Difficult airway management encountered in an unconscious patient. Can. J. Anesth. J. Can. D’anesthésie.

[12] Frerk C., Mitchell V.S., McNarry A.F., Mendonca C., Bhagrath R., Patel A., O’Sullivan E.P., Woodall N.M., Ahmad I. (2015). Difficult Airway Society 2015 guidelines for management of unanticipated difficult intubation in adults. Br. J. Anaesth..

[13] Doyle D.J. (2004). Awake intubation using the GlideScope video laryngoscope: Initial experience in four cases. Can. J. Anaesth..

[14] Kramer A., Müller D., Pförtner R., Mohr C., Groeben H. (2015). Fibreoptic vs videolaryngoscopic (C-MAC(®) D-BLADE) nasal awake intubation under local anaesthesia. Anaesthesia.

[15] Rosenstock C.V., Thøgersen B., Afshari A., Christensen A.L., Eriksen C., Gätke M.R. (2012). Awake fiberoptic or awake video laryngoscopic tracheal intubation in patients with anticipated difficult airway management: A randomized clinical trial. Anesthesiology.

[16] Noppens R.R., Geimer S., Eisel N., David M., Piepho T. (2012). Endotracheal intubation using the C-MAC(R) video laryngoscope or the Macintosh laryngoscope: A prospective, comparative study in the ICU. Crit. Care.

[17] Abdellatif A.A., Ali M.A. (2014). GlideScope videolaryngoscope versus flexible fiberoptic bronchoscope for awake intubation of morbidly obese patient with predicted difficult intubation. Middle East J. Anaesthesiol..

[18] Wahba S.S., Tammam T.F., Saeed A.M. (2012). Comparative study of awake endotracheal intubation with Glidescope video laryngoscope versus flexible fiber optic bronchoscope in patients with traumatic cervical spine injury. Egypt. J. Anaesth..

[19] von Elm E., Altman D.G., Egger M., Pocock S.J., Gøtzsche P.C., Vandenbroucke J.P. (2007). Strengthening the Reporting of Observational Studies in Epidemiology (STROBE) statement: Guidelines for reporting observational studies. BMJ.

[20] Turkstra T.P., Harle C.C., Armstrong K.P., Armstrong P.M., Cherry R.A., Hoogstra J., Jones P.M. (2007). The GlideScope-specific rigid stylet and standard malleable stylet are equally effective for GlideScope use. Can. J. Anesth..

[21] El-Boghdadly K., Pawa A., Chin K.J. (2018). Local anesthetic systemic toxicity: Current perspectives. Local. Reg. Anesth..

[22] Ramsay M.A., Savege T.M., Simpson B.R., Goodwin R. (1974). Controlled sedation with alphaxalone-alphadolone. Br. Med. J..

[23] Jaber S., De Jong A., Pelosi P., Cabrini L., Reignier J., Lascarrou J.B. (2019). Videolaryngoscopy in critically ill patients. Crit. Care.

[24] Perbet S., De Jong A., Delmas J., Futier E., Pereira B., Jaber S., Constantin J.M. (2015). Incidence of and risk factors for severe cardiovascular collapse after endotracheal intubation in the ICU: A multicenter observational study. Crit. Care.

[25] Lascarrou J.B., Boisrame-Helms J., Bailly A., Le Thuaut A., Kamel T., Mercier E., Ricard J.D., Lemiale V., Colin G., Mira J.P. (2017). Video Laryngoscopy vs Direct Laryngoscopy on Successful First-Pass Orotracheal Intubation Among ICU Patients: A Randomized Clinical Trial. JAMA.

[26] Cabrini L., Landoni G., Baiardo Redaelli M., Saleh O., Votta C.D., Fominskiy E., Putzu A., Snak de Souza C.D., Antonelli M., Bellomo R. (2018). Tracheal intubation in critically ill patients: A comprehensive systematic review of randomized trials. Crit. Care.

[27] Mosier J.M., Whitmore S.P., Bloom J.W., Snyder L.S., Graham L.A., Carr G.E., Sakles J.C. (2013). Video laryngoscopy improves intubation success and reduces esophageal intubations compared to direct laryngoscopy in the medical intensive care unit. Crit. Care.

[28] De Jong A., Molinari N., Conseil M., Coisel Y., Pouzeratte Y., Belafia F., Jung B., Chanques G., Jaber S. (2014). Video laryngoscopy versus direct laryngoscopy for orotracheal intubation in the intensive care unit: A systematic review and meta-analysis. Intensive Care Med..

[29] Xue F.S., Zhang G.H., Liu J., Li X.Y., Yang Q.Y., Xu Y.C., Li C.W. (2007). The clinical assessment of Glidescope in orotracheal intubation under general anesthesia. Minerva Anestesiol..

[30] Aghamohammadi H., Massoudi N., Fathi M., Jaffari A., Gharaei B., Moshki A. (2015). Intubation learning curve: Comparison between video and direct laryngoscopy by inexperienced students. J. Med. Life.

[31] Sakles J.C., Mosier J., Patanwala A.E., Dicken J. (2014). Learning curves for direct laryngoscopy and GlideScope(R) video laryngoscopy in an emergency medicine residency. West. J. Emerg. Med..

[32] Ahmad I., El-Boghdadly K., Bhagrath R., Hodzovic I., McNarry A.F., Mir F., O’Sullivan E.P., Patel A., Stacey M., Vaughan D. (2020). Difficult Airway Society guidelines for awake tracheal intubation (ATI) in adults. Anaesthesia.

[33] Moore A., El-Bahrawy A., El-Mouallem E., Lattermann R., Hatzakorzian R., LiPishan W., Schricker T. (2017). Videolaryngoscopy or fibreoptic bronchoscopy for awake intubation of bariatric patients with predicted difficult airways—A randomised, controlled trial. Anaesthesia.

[34] Fitzgerald E., Hodzovic I., Smith A.F. (2015). ‘From darkness into light’: Time to make awake intubation with videolaryngoscopy the primary technique for an anticipated difficult airway?. Anaesthesia.

[35] Mahran E.A.E.-H., Hassan M.E. (2016). Comparative randomised study of GlideScope(^®^) video laryngoscope versus flexible fibre-optic bronchoscope for awake nasal intubation of oropharyngeal cancer patients with anticipated difficult intubation. Indian J. Anaesth..

[36] Mendonca C., Mesbah A., Velayudhan A., Danha R. (2016). A randomised clinical trial comparing the flexible fibrescope and the Pentax Airway Scope (AWS)(^®^) for awake oral tracheal intubation. Anaesthesia.

[37] Fiadjoe J.E., Litman R.S. (2012). Difficult tracheal intubation: Looking to the past to determine the future. Anesthesiology.

[38] Lakticova V., Koenig S.J., Narasimhan M., Mayo P.H. (2013). Video Laryngoscopy is Associated With Increased First Pass Success and Decreased Rate of Esophageal Intubations During Urgent Endotracheal Intubation in a Medical Intensive Care Unit When Compared to Direct Laryngoscopy. J. Intensive Care Med..

[39] Kory P., Guevarra K., Mathew J.P., Hegde A., Mayo P.H. (2013). The impact of video laryngoscopy use during urgent endotracheal intubation in the critically ill. Anesth. Analg..

[40] Cook T.M., Woodall N., Frerk C. (2011). Major complications of airway management in the UK: Results of the Fourth National Audit Project of the Royal College of Anaesthetists and the Difficult Airway Society. Part 1: Anaesthesia. Br. J. Anaesth..

[41] Sakles J.C., Mosier J.M., Chiu S., Keim S.M. (2011). Tracheal Intubation in the Emergency Department: A Comparison of GlideScope((R)) Video Laryngoscopy to Direct Laryngoscopy in 822 Intubations. J. Emerg. Med..

[42] Sakles J.C., Mosier J., Chiu S., Cosentino M., Kalin L. (2012). A comparison of the C-MAC video laryngoscope to the Macintosh direct laryngoscope for intubation in the emergency department. Ann. Emerg. Med..

[43] Shamim F., Sohaib M., Samad K., Khan M.F., Manji A.A., Latif A. (2023). Ease of Intubation with McGrath Videolaryngoscope and Incidence of Adverse Events During Tracheal Intubation in COVID-19 Patients: A Prospective Observational Study. J. Crit. Care Med..

[44] Cortellazzi P., Caldiroli D., Byrne A., Sommariva A., Orena E.F., Tramacere I. (2015). Defining and developing expertise in tracheal intubation using a GlideScope((R)) for anaesthetists with expertise in Macintosh direct laryngoscopy: An in-vivo longitudinal study. Anaesthesia.

[45] Simpson G.D., Ross M.J., McKeown D.W., Ray D.C. (2012). Tracheal intubation in the critically ill: A multi-centre national study of practice and complications. Br. J. Anaesth..

[46] Kleine-Brueggeney M., Buttenberg M., Greif R., Nabecker S., Theiler L. (2017). Evaluation of three unchannelled videolaryngoscopes and the Macintosh laryngoscope in patients with a simulated difficult airway: A randomised, controlled trial. Anaesthesia.

[47] Lewis S.R., Butler A.R., Parker J., Cook T.M., Schofield-Robinson O.J., Smith A.F. (2017). Videolaryngoscopy versus direct laryngoscopy for adult patients requiring tracheal intubation: A Cochrane Systematic Review. Br. J. Anaesth..

[48] Pieters B.M.A., Maas E.H.A., Knape J.T.A., van Zundert A.A.J. (2017). Videolaryngoscopy vs. direct laryngoscopy use by experienced anaesthetists in patients with known difficult airways: A systematic review and meta-analysis. Anaesthesia.

[49] Hoshijima H., Mihara T., Maruyama K., Denawa Y., Takahash M., Shiga T., Nagasaka H. (2018). McGrath videolaryngoscope versus Macintosh laryngoscope for tracheal intubation: A systematic review and meta-analysis with trial sequential analysis. J. Clin. Anesth..

[50] Janz D.R., Casey J.D., Semler M.W., Russell D.W., Dargin J., Vonderhaar D.J., Dischert K.M., West J.R., Stempek S., Wozniak J. (2019). Effect of a fluid bolus on cardiovascular collapse among critically ill adults undergoing tracheal intubation (PrePARE): A randomised controlled trial. Lancet Respir. Med..

[51] De Jong A., Myatra S.N., Roca O., Jaber S. (2022). How to improve intubation in the intensive care unit. Update on knowledge and devices. Intensive Care Med..

